# Epidemic of plague amidst COVID-19 in Madagascar: efforts, challenges, and recommendations

**DOI:** 10.1186/s41182-021-00349-5

**Published:** 2021-07-13

**Authors:** Abdullahi Tunde Aborode, Ana Carla dos Santos Costa, Anmol Mohan, Samarth Goyal, Aishat Temitope Rabiu, Christos Tsagkaris, Olivier Uwishema, Oumaima Outani, Shoaib Ahmad, Mohammad Yasir Essar

**Affiliations:** 1 Healthy Africans Platform, Research and Development, Ibadan, Nigeria; 2 Federal University of Bahia, Salvador, Bahia Brazil; 3 Karachi Medical & Dental College, Karachi, Pakistan; 4 KMC Manipal, Manipal, India; 5 Faculty of Basic Medical Sciences, Kwara State University, Malete, Nigeria; 6 University of Crete, Faculty of Medicine, Heraklion, Greece; 7 Oli Health Magazine Organization, Research and Education, Kigali, Rwanda; 8 Faculty of Medicine, Karadeniz Technical University, 61080 Trabzon, Turkey; 9 Clinton Global Initiative University, New York, USA; 10 Faculty of Medicine and Pharmacy of Rabat, Mohamed 5 University, Rabat, Morocco; 11 District Head Quarters Teaching Hospital, Faisalabad, Pakistan; 12 Kabul University of Medical Sciences, Kabul, Afghanistan

**Keywords:** Plague, Slow, Epidemic, COVID-19, Pandemic, Madagascar

## Abstract

The plague has been wreaking havoc on people in Madagascar with the COVID-19 pandemic. Madagascar’s healthcare sector is striving to respond to COVID-19 in the face of a plague outbreak that has created a new strain on the country’s public health system. The goal and activities of the gradual epidemic of plague in Madagascar during COVID-19 are described in this research. In order to contain the plague and the COVID-19 pandemic in this country, we have suggested long-term recommendations that can help to contain the outbreak so that it may spread to non-endemic areas.

## Introduction

The plague has afflicted humans for centuries, causing three major pandemics that resulted in 200 million deaths during the 1990s years [1]. The disease, caused by *Yersinia pestis*, a bacterium that normally lives in a rodent host and flea vector, has been prevalent in the Madagascar highlands, above 800 m altitudes, since the 1920s. Therefore, plague is endemic in Madagascar, where 200 to 700 cases are reported every year, primarily in the bubonic form, which outbreaks follow a seasonal trend [2–4].

From January 1 to March 11, 2021, at least 21 confirmed cases of bubonic plague have been confirmed in Madagascar [5]. Eight of these cases have been reported since March 1, 2021, in Ambositra and Mandarina [5]. Since the start of the year 2021, 37 suspected cases have been reported, affecting multiple regions, including Alaotra-Mangoro, Analamanga, Haute Matsiatra, and Itasy [5]. Until 12 March 2021, the disease led to approximately nine deaths [5].

## Plague and COVID-19 pandemic in Madagascar

Currently, the main risk people face is the occurrence of an outbreak of plague among humans in the midst of the COVID-19 pandemic. This occurrence is due to the poor state of the country’s public health system and limited access to health in rural areas; these factors were aggravated by the COVID-19 pandemic. The main problem of the COVID-19 outbreak now would be the coexistence of two potentially fatal diseases. Bubonic plague, for example, has a case fatality rate of 20.8% with treatment; in countries like Madagascar, where superstitions, lack of funding, and distance from medical facilities can delay access to health care, the mortality rate can reach 40–70% without treatment [6, 7].

Furthermore, the disease can also spread from person to person through airborne droplets, causing pneumonic plague [8], which causes shortness of breath and cough, symptoms that are also found on COVID-19 infection and can lead to misdiagnosis. In fact, misdiagnosis of infectious diseases due to the overlap of symptoms with COVID-19 was already related in other countries, such as Brazil, India, and Pakistan [9–12]. The main problem of this misdiagnosis is that the fatality rate of pneumonic plague, which is 60.5% with treatment, can reach 100% without appropriate management [6, 7].

In fact, a high incidence of pneumonic plague was observed in recent years in Madagascar. From August 1 to November 10, 2017, the pneumonic plague was diagnosed in 1618 cases and 72 deaths were registered in Madagascar [7]. The number of cases, however, is almost certainly much higher due to inadequate diagnostic services and underreporting. For example, among those 1618 cases, only 365 (23%) were confirmed, 573 (35%) were probable cases, and 680 (42%) were suspected cases in Madagascar [7].

Thus, the overlap of symptoms between pneumonic plague and COVID-19, leading to diagnostic delay, and the increase in the number of bubonic plagues can be a dangerous combination to the Madagascar health system. Madagascar and other African countries such as the Democratic Republic of the Congo, Mozambique, Uganda, and Tanzania are particularly vulnerable to plagues and are the most burdened with the endemic of plague in the world [13, 14], due to the close proximity due to the poor rural communities and high consumption of insufficiently cooked meat of infected animals [15]. However, factors like climate change, unconserved and low sanitation in the environment, poverty, urbanization, and migration increase the burden and further spread of plague disease making it transmittable in unaffected areas [16]. In addition to plague, some African countries faced other infectious diseases and viral outbreaks such as bird flu, malaria, Ebola, and measles [17–20]. To prevent the devastating scenario that is brought about by the presence of a public health system, it is poorly organized and equipped, whose reforms are hampered by political and social instability, and urgent measures are needed as soon as possible.

## Efforts

The Ministry of Health in Madagascar proposed a National Plague Control Program. The program has two components [13, 14]:

- Case surveillance, based on immediate notification of suspected cases, followed by treatment with antibiotics if found positive.
- Vector (flea) is controlled by increased insecticide spraying at workplaces and residential areas [7, 8, 21].

However, severe economic limitations, such as the disparity in resources in rural areas as compared to urban and limited testing, have been major challenges in the execution of this ideal program [22, 23].

Furthermore, economic resources need to be strengthened before this program can live up to its full potential. In response to the outbreak, the World Health Organization (WHO) quickly released US$ 1.5 million in emergency funds, distributed over 1.2 million antibiotic doses, and trained over 4400 people to serve as “touch tracers” to help prevent the disease from spreading further in hard-hit areas [13].

In synergy with the WHO initiative, Madagascar needs to reallocate more funds to the healthcare sector to facilitate prompt detection, isolation, and treatment of the suspected cases and to prevent and contain other outbreaks. With the aid of WHO and other organizations, Madagascar’s Ministry of Public Health has organized the health response and triggered crisis units in Antananarivo and Toamasina, where all cases and contacts have been given free care or prophylactic antibiotics [14]. More such healthcare benefits need to be introduced to decrease the burden of the disease.

## Conclusion and recommendations

In rural areas, a lack of laboratory facilities for the biological diagnosis of plague remains a significant impediment to early diagnosis. The isolation of *Y. pestis* has been the key confirmatory test so far (requiring a minimum of 4 days). Rapid diagnostic tests, which are now considered a validation tool in endemic areas, have recently been developed, opening new possibilities in terms of surveillance and case management, which must be expanded in remote centers [5, 8].

Extensive public health response measures need to be implemented. Increased awareness among the general population through campaigns, posters, and social media about the signs, symptoms, prevention, and infection control during the burial of the deceased would lead to a significant reduction in cases. In addition to this, healthcare workers need to be trained on the improved ways of case detection, infection control measures, and personal protection from the spreading disease.

Along with controlling the spread of indigenous infection, care must be taken to prevent the spread to adjoining territories. Strengthening the exit screening at airports and ports to reduce the risk of international spread should be effective. Because of their trade and travel ties to Madagascar, 9 African countries and overseas territories (Comoros, Ethiopia, Kenya, Mauritius, Mozambique, La Réunion (France), Seychelles, South Africa, and Tanzania) have been listed as priority countries for plague preparedness and readiness. These countries are implementing preparedness measures such as raising public awareness about the plague, improving disease surveillance (particularly at points of entry), and stockpiling equipment and supplies [24].

The plague outbreak cases in Madagascar are slowing, but the response must continue [5]. The epidemic’s darkest days are behind us, but as the plague season in Madagascar ends, the ability to diagnose and respond to new infections becomes a burden. Since the number of new infections has been steadily declining in recent weeks, this means that the epidemic has been contained, but more bubonic and pneumonic plague infections are anticipated. Even though the disease is endemic to Madagascar, the outbreak’s pace is unparalleled, and it may spread to non-endemic areas.

## Data Availability

Not applicable

## Abbreviations

COVID-19: Coronavirus disease 2019
US: United States of America
