# Correction to: Epidemic of plague amidst COVID-19 in Madagascar: efforts, challenges, and recommendations

**DOI:** 10.1186/s41182-021-00352-w

**Published:** 2021-07-29

**Authors:** Abdullahi Tunde Aborode, Ana Carla dos Santos Costa, Anmol Mohan, Samarth Goyal, Aishat Temitope Rabiu, Christos Tsagkaris, Olivier Uwishema, Oumaima Outani, Shoaib Ahmad, Mohammad Yasir Essar

**Affiliations:** 1 Healthy Africans Platform, Research and Development, Ibadan, Nigeria; 2 Federal University of Bahia, Salvador, Bahia Brazil; 3 Karachi Medical & Dental College, Karachi, Pakistan; 4 KMC Manipal, Manipal, India; 5 Faculty of Basic Medical Sciences, Kwara State University, Malete, Nigeria; 6 University of Crete, Faculty of Medicine, Heraklion, Greece; 7 Oli Health Magazine Organization, Research and Education, Kigali, Rwanda; 8 Faculty of Medicine, Karadeniz Technical University, 61080 Trabzon, Turkey; 9 Clinton Global Initiative University, New York, USA; 10 Faculty of Medicine and Pharmacy of Rabat, Mohamed 5 University, Rabat, Morocco; 11 District Head Quarters Teaching Hospital, Faisalabad, Pakistan; 12 Kabul University of Medical Sciences, Kabul, Afghanistan

**Correction to: Trop Med Health 49, 56 (2021)**

**https://doi.org/10.1186/s41182-021-00349-5**

Following publication of the original article [1], the affiliation numbers for the authors Oumaima Outani, Shoaib Ahmad, and Mohammad Yasir Essar were incorrect, and should be changed from Oumaima Outani^9,10^, Shoaib Ahmad^10,11^ and Mohammad Yasir Essar^11,12^ to Oumaima Outani^10^, Shoaib Ahmad^11^ and Mohammad Yasir Essar^12^.

The author affiliation list has been updated above and the original article [1] has been corrected.

## References

[1] Aborode (2021). Epidemic of plague amidst COVID-19 in Madagascar: efforts, challenges, and recommendations. Trop Med Health.

